# What Is the Outlier—Consistent Outlier or Inconsistent Outlier?

**DOI:** 10.1002/ansa.70030

**Published:** 2025-07-24

**Authors:** Hiromasa Kaneko

**Affiliations:** ^1^ Department of Applied Chemistry School of Science and Technology Meiji University Kawasaki Kanagawa Japan

## Abstract

In the design of molecules, materials and processes, outliers or outlier samples can be included in a dataset when performing machine learning or regression analysis. Although outlier samples with high prediction errors in regression analysis have been divided into bad leverage points and vertical outliers (good leverage points have low prediction errors), this study classifies the outlier samples into consistent outliers (CO) and inconsistent outliers (ICO) for a detailed discussion of outlier samples and their effective utilisation. The relationship between the explanatory variables (*x*) and dependent variables (*y*) is consistent with the other samples for CO but not for ICO. Furthermore, an index of ICO‐likeness based on triple cross‐validation and the mean absolute error is proposed, and a method to determine whether an outlier sample is an ICO or a CO is developed. Data analysis using numerical simulation datasets and a compound dataset with boiling points confirms that the proposed method can appropriately discriminate between ICO and CO. When an outlier sample is determined to be an ICO, the errors in *x* and *y* should be checked first for the sample. If no errors exist in *x* and *y*, a new *x* should be added to explain *y* of the ICO. When an outlier sample is determined to be CO, it is expected that exploring the extrapolation from CO in *x* will further improve the *y* values using a model that includes CO.

## 1 Introduction

In molecular design, material design, process design and process control, a mathematical model *y* = *f*(*x*) is constructed between features *x*, such as molecular descriptors, experimental conditions, synthesis conditions, manufacturing conditions, evaluation conditions, process conditions and process variables, and dependent variables *y*, such as the properties and activities of molecules, materials and product quality, with a dataset using machine learning. The model is used to predict *y* values by inputting *x* values into the model *y* = *f*(*x*) or by designing *x* values for which *y* has target values.

Outliers may exist in a dataset. Because outliers reduce the predictive ability of a model, it is important to detect them appropriately. Xu et al. proposed a time‐series Kalman filter that handles outlier detection for time‐series data of dynamic systems, where normal process changes often mask the existence of outliers [1]. Cao et al. proposed an ensemble partial least squares regression for descriptor selection, outlier detection and applicability domain in QSAR/QSPR modelling [2]. In another study, Dai and Genton proposed visualising both the magnitude and shape outlyingness of multivariate functional data for data visualisation and outlier detection [3]. Kaneko proposed automatic detection based on regression analysis and repeated ensemble learning for outlier samples rather than outlier data [4]. To identify shear transformation zones, Liang et al. introduced a consistent, automated and robust method using linear‐based machine learning outlier detection algorithms [5]. Todkar et al., meanwhile, proposed a one‐class support vector machine‐based outlier detection approach to detect A‐scan data vectors that differ from a reference dataset collected over a known healthy pavement area [6]. Ouyang et al. proposed an unsupervised ensemble‐based outlier detection approach that considers the union of different outlier detection algorithms, wherein each of the selected detectors is only responsible for identifying the small number of outliers that are most obvious from their respective standpoints [7]. Moreover, Rhyu et al. proposed an approach for automatically detecting outliers using principal component analysis and contributions and estimating missing data using various general‐purpose algorithms whilst reducing the impact of outliers [8]. Puchhammer and Filzmoser proposed an outlier detection method that accounts for a continuously varying covariance structure depending on the spatial neighbourhood of observations [9]. In their study, Murph et al. extended several methods for exploratory data analysis of probability density functions and compared them to simulated data that exhibited different types of variation, designed to mimic those observed in real‐world applications, and then used methods to perform exploratory data analysis of the breakthrough curves observed in gas transport simulations for underground fracture networks [10]. Brownfield and Kalivas separately detected both spectral outliers for *x* (*x*‐outlier) and analyte outliers for *y* (*y*‐outlier) using partial least squares regression and the sum of the ranking differences in a spectral analysis [11].

This study deals with outlier samples, including *x*‐, *y*‐, or *x*‐ and *y*‐outliers. However, it does not discuss outlier sample detection but rather the classification and utilisation of outlier samples. Therefore, it is assumed that the outlier samples have already been detected using outlier detection methods, as in the aforementioned references. Outlier samples have low similarity to other samples and different *x* and *y* relationships compared to other samples; thus, outlier samples can contain considerable information unless they are data anomalies, such as experimental errors and transcription errors. To discuss outlier samples in detail in this study, outlier samples are classified into two categories:

- Consistent outlier (CO)
- Inconsistent outlier (ICO)

**FIGURE 1:**
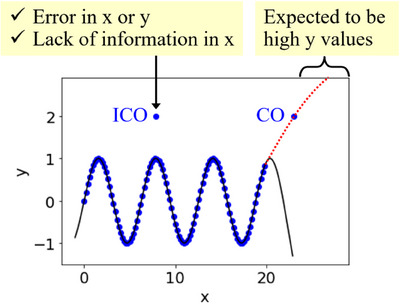
Basic concept of CO and ICO. Blue points and black line indicate training data and regression model, respectively, and the way to handle the outlier sample after it is determined to be ICO or CO.

The concepts of CO and ICO are illustrated in Figure 1. The regression model in Figure 1 is a relationship between *x* and *y* constructed using the current samples, and it does not necessarily represent the true relationship between *x* and *y*. The current regression model is unable to express the two outliers in Figure 1. The CO sample can explain *y* with the current *x* by improving the regression model without changing *x* itself, and thus, it is called CO (consistent outlier) because it is consistent with other samples, including the outlier sample. Although CO is an outlier sample and cannot be explained by a model constructed with training data other than CO, the relationship between *x* and *y* is consistent with the other samples or is a distant extension of that relationship, as indicated by the red dotted line in Figure 1. Appropriate model construction using training data may explain CO, and increasing the sample size increases the likelihood of explaining it. On the other hand, *y* of the ICO sample cannot be explained with only the current *x*, no matter how the regression model is changed, and thus, it is called ICO (inconsistent outlier). Because ICO is an outlier sample, having a different relationship with *x* and *y* than with *x* and *y* in the other samples, as shown in Figure 1, the current *x* cannot explain the *y* value of the ICO using this model. To construct a regression model with ICO, it is necessary to add new variables to *x* that can explain ICO.

As explained in Figure 1, the model construction that considers the outlier sample varies completely depending on the type of outlier sample. Therefore, this study aims to determine whether the outlier samples are CO or ICO. To detect outlier samples, the prediction errors of the samples with the model constructed using the other samples were used, and the samples with large prediction errors were determined to be outlier samples. However, even if an outlier sample can be detected using this method, it is not possible to determine whether the outlier sample is a CO or ICO.

CO and ICO are completely different from the terms ‘good leverage points’, ‘vertical outliers’ and ‘bad leverage points’ [12]. On one hand, ‘vertical outliers’ and ‘bad leverage points’ have high prediction errors and ‘good leverage points’ have low prediction errors in regression analysis, and on the other hand, both ICO and CO have high prediction errors and are classified for a detailed discussion of outlier samples and their effective utilisation. Whilst ‘good leverage points’, ‘vertical outliers’ and ‘bad leverage points’ are used to classify outliers in outlier detection, ICO and CO are used to classify outliers after outlier detection with the aim of reconstructing a regression model and conducting predictions using the model. Furthermore, the proposed method is also different from outlier detection. For example, even when a sample is detected as a ‘good leverage point’, ‘vertical outlier’ or ‘bad leverage point’, there exists no idea whether the outlier sample should be used to improve the regression model or whether the regression model is reconstructed including the outlier sample and prediction is conducted with the model. In some studies, researchers straightforwardly remove outlier samples, which leads to the loss of useful information. By classifying the outlier sample as ICO or CO after outlier detection, we can answer the question of what to do with the regression model by using outlier samples.

This study focused on prediction errors when predicting samples in models constructed with and without outlier samples. Various methods exist to calculate prediction errors, including training and test data division and cross‐validation variants [13, 14]. In this study, prediction errors were calculated based on the concept of double cross‐validation (DCV) [15, 16]. DCV corresponds to repeatedly performing training and test data division, and it is possible to calculate prediction errors for complete test data, regardless of the number of outer folds. Prediction errors when predicting samples using the model constructed with the outlier sample are represented as prediction errors of the model constructed with the outlier sample (PEwOS), and those without the outlier sample are represented as prediction errors of the model constructed without the outlier sample (PEwoOS). When PEwOS and PEwoOS are close, or PEwOS is lower than PEwoOS, the relationship between *x* and *y*, including the outlier sample and other samples, is consistent, and the outlier sample is considered CO. When PEwOS is higher than PEwoOS, the relationship between *x* and *y* for the outlier sample is different from that for the other samples, and the outlier sample is considered an ICO.

Using numerical simulations and compound datasets, each outlier sample was detected, and it was determined whether the outlier sample was CO or ICO using the proposed method. Samples determined to be CO or ICO are discussed to verify the effectiveness of the proposed method.

## 2 Method

One method of detecting outlier samples is to calculate the prediction error for each sample. This method can detect samples with large prediction errors as outlier samples; however, it cannot determine whether the outlier sample is a CO or ICO. Therefore, we focused on PEwOS and PEwoOS in this study. If PEwOS is higher than PEwoOS, the outlier sample has a larger impact on the model, and we can determine that the outlier sample is an ICO. However, if the difference between PEwOS and PEwoOS is small, or PEwOS is lower than PEwoOS, the relationship between *x* and *y* of the outlier sample and other samples is considered consistent and can be determined as CO.

**FIGURE 2:**
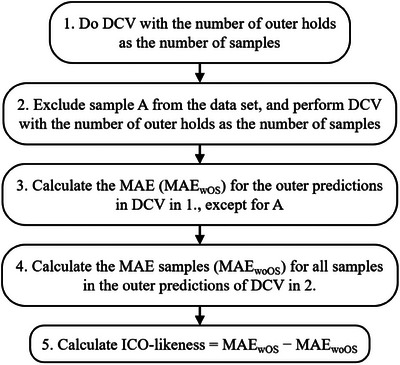
Flowchart of calculating ICO‐likeness.

Figure 2 shows the flowchart of calculating ICO‐likeness and the algorithm of the proposed method is given as follows:

1. Do DCV with the number of outer holds as the number of samples, which implies leave‐one‐out (DCV is considered a cross‐validation method for regression analysis without hyperparameters).
2. Exclude Sample A from the dataset, and perform DCV with the number of outer holds as the number of samples (the number of samples in this dataset 2 is less than that in the dataset in 1).
3. Calculate the mean absolute error (MAE) for the outer predictions in DCV in 1, except for A. This is denoted as MAE in DCV with outlier samples (MAE_wOS_).
4. Calculate the MAE for all samples in the outer predictions of DCV in 2. This is denoted as MAE in DCV without outlier samples (MAE_woOS_).
5. Calculate the ICO‐likeness as follows:
(1) $$\mathrm{ICO}-\mathrm{likeness}=\mathrm{MA}E_{\mathrm{wOS}}-\mathrm{MA}E_{\mathrm{woOS}}$$

where, the samples for calculating MAE_wOS_ and MAE_woOS_ are identical, and the MAE can be simply compared. When the ICO‐likeness value is large compared with ICO‐likeness for normal samples that are not outlier samples, implying that the prediction accuracy decreases by including Sample A, Sample A is excluded from 2, which are considered to have a different relationship between *x* and *y* than the other samples, and Sample A is considered an ICO. However, when the ICO‐likeness value is low, Sample A is excluded from 2 and is considered to have a consistent relationship between *x* and *y* with the other samples. In contrast, Sample A is considered CO.

By changing Sample A to another sample in the dataset and conducting Steps 2–6, the ICO‐likeness can be calculated for any sample, implying a triple cross‐validation. Although ICO‐likeness can be calculated for all samples when the number of samples is large, calculation time is required; thus, ICO‐likeness is calculated only for those samples for which the absolute errors between the actual *y* values and those predicted in the DCV are large. Furthermore, the computation time can be further shortened by setting the number of holds outside the DCV equal to 2, smaller than the number of samples, that is, 2, 5 and 10. In this case, the outer division of DCV in 1 should be the same as that of the outer division in the DCV in 2. In addition, Sample A should always be included in the training data at the time of DCV in 1.

MAE is calculated for prediction errors of the model constructed with outlier samples and those of the model constructed without outlier samples. Even in the latter case, although an outlier sample is included when the model is constructed, the samples used to calculate MAE do not include any outliers. Therefore, sensitivity to outliers is not required, and the MAE is used to calculate the average of the predictions.

Python codes for the proposed method are available at https://github.com/hkaneko1985/dcekit.

Figure 1 shows the method for handling an outlier sample after it is determined to be an ICO or CO. When determined to be ICO, the relationship between *x* and *y* in the ICO and the other samples is not consistent; therefore, errors in *x* and *y* should first be checked. When neither *x* nor *y* is wrong, the current *x* cannot explain *y*. Therefore, it is necessary to consider a new *x* to explain *y* for the ICO. Researchers can examine ICO samples in relation to other samples with similar *x* values but substantially different *y* values. From this comparison, one may explore candidate variables that can discriminate between the ICO sample and these similar‐yet‐different samples, possibly by consulting domain knowledge, performing feature construction or feature engineering.

When the sample is determined to be CO, further exploration of the extrapolation from CO is expected to improve the *y* value over the existing *y* values because the relationship between *x* and *y* in CO and the other samples is consistent, and CO is an outlier compared to the other *y* values. Figure 1 shows the case where the *y* value of CO is large. When the *y* value of CO is small, further search for extrapolation from CO is expected to further reduce the *y* value from the existing *y* values. As previously described, the response differs after an outlier sample is determined to be an ICO or CO.

The proposed method is based on the assumption that outlier samples have already been detected. The proposed method can classify whether the outlier sample is ICO and new variables should be added to *x*, or whether the outlier sample is CO and the model is reconstructed including CO. When there exist multiple outlier samples detected in advance, all outliers are removed from the dataset first, and then, the proposed method can classify each outlier as either CO or ICO whilst adding each outlier sample to the dataset one by one.

## 3 Results and Discussion

**FIGURE 3:**
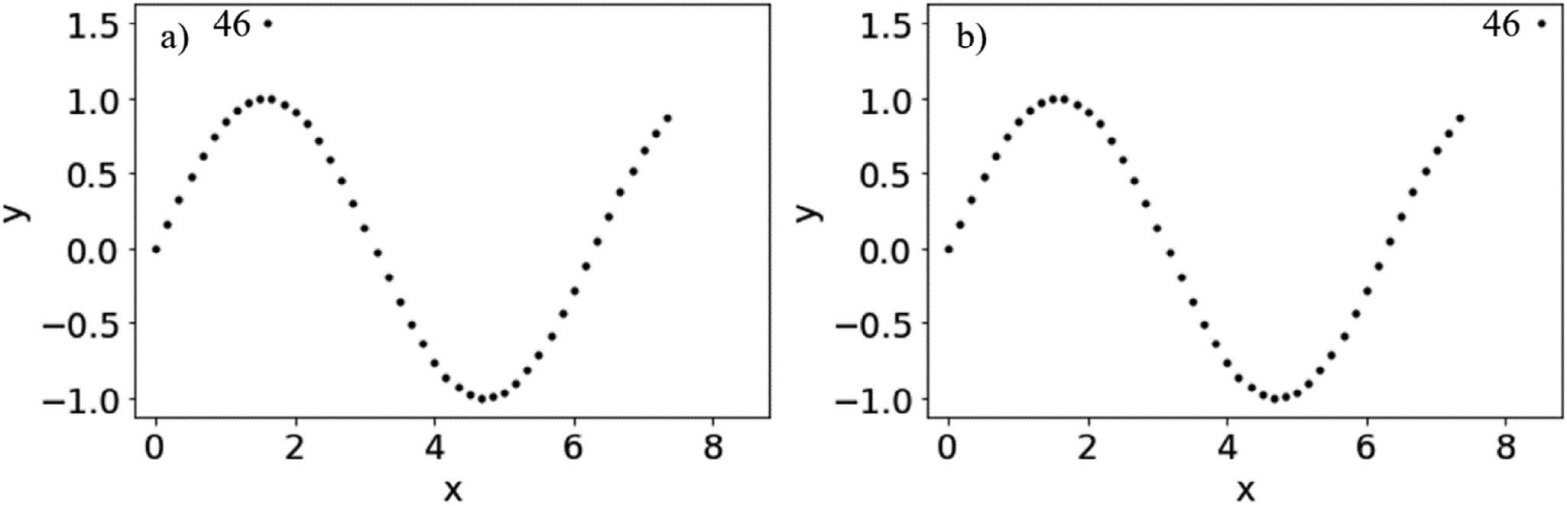
Relationship between *x* and *y* in the numerical simulation datasets. (a) Dataset 1, (b) Dataset 2.

To test the performance of the proposed ICO‐likeness, a nonlinear function of *x* and *y* was first assumed to be a system in which *y* was obtained through experimentation based on *x*. The nonlinear function was based on the sine curve and is expressed as follows:

(2) $$y=\sin\left(x\right)$$

where, *z* was 0, 1, …, 44, and *x* was z/6. *y* was calculated using Equation (1), and 45 *x* and *y* samples are generated. Then, one outlier sample was generated in two ways and added to the 45 samples in Datasets 1 and 2. The relationships between *x* and *y* for Datasets 1 and 2 are shown in Figure 3. Although the 46th sample in Figure 3a indicates ICO and the 46th sample in Figure 3b indicates CO, the following data analysis was performed with these factors unknown.

Gaussian process regression (GPR) [17] was used as the regression analysis method. The kernel function of the GPR used in this study is as follows:

(3) $$K\left(x^{\left(i\right)},x^{\left(j\right)}\right)=\theta_{0}\exp\left\{-\frac{\theta_{1}}{2}{\left\|x^{\left(i\right)}-x^{\left(j\right)}\right\|}^{2}\right\}+\theta_{2}$$

where **x** ^(^ *^i^* ^)^ is the *i*th sample of *x*, and *θ*_0_, *θ*_1_ and *θ*_2_ are the hyperparameters, which were optimised in the scikit‐learn program.

**FIGURE 4:**
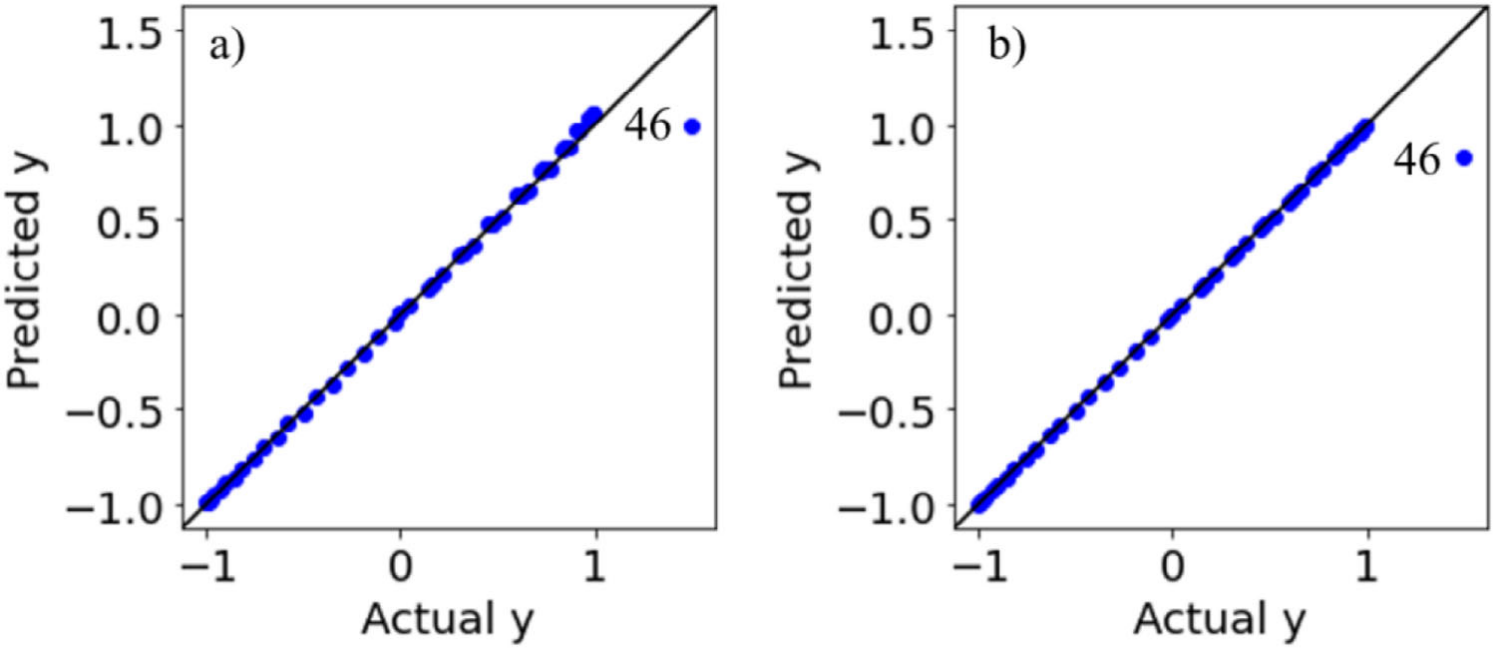
Results of DCV in the numerical simulation datasets. (a) Dataset 1, (b) Dataset 2.

**FIGURE 5:**
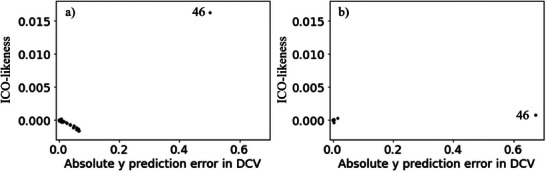
Plots of absolute prediction error of *y* in DCV and ICO‐likeness in the numerical simulation datasets. (a) Dataset 1, (b) Dataset 2.

The results of the leave‐one‐out cross‐validation for Datasets 1 and 2 are shown in Figure 4. The absolute prediction errors for both the 46th sample in Dataset 1 and the 46th sample in Dataset 2 were large. Although we recognise these as outlier samples, we cannot determine whether they are ICO or CO, as shown in Figure 3. Plots of the absolute prediction error of *y* in DCV and ICO‐likeness for Datasets 1 and 2 are shown in Figure 5. Compared with the other samples, the ICO‐likeness values of the 46th sample in Dataset 1 were large, indicating that the sample was an ICO. In contrast, the ICO‐likeness value for the 46th sample in Dataset 2 was small, even though the absolute prediction error was large. Thus, the sample was determined to be CO. Because the 46th samples in Datasets 1 and 2 were actually ICO and CO, respectively, Figure 5 confirms that the proposed ICO‐likeness can determine whether the outlier samples are ICO or CO.

A dataset of boiling points [18] was used. RDKit [19] was used to calculate the molecular descriptors. RDKit afforded 2D descriptors, such as the number of atoms for each atom type, molecular weight and descriptors, including information on fragments, topology and physicochemical properties, where *y* is the boiling point, and *x* is the molecular descriptor.

**FIGURE 6:**
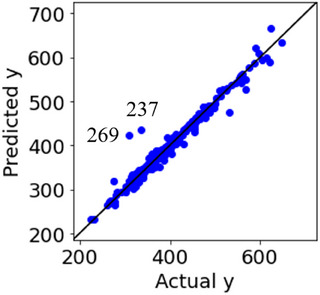
Results of DCV in the compound dataset.

**FIGURE 7:**
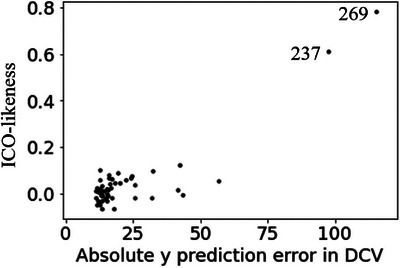
Plot of absolute prediction error of *y* in DCV and ICO‐likeness in the compound dataset.

The results of the leave‐one‐out cross‐validation in the GPR are shown in Figure 6. Several samples, especially 237th and 269th, had large absolute *y* prediction errors in DCV. Figure 7 shows the results of calculating ICO‐likeness for 50 samples with large absolute *y* prediction errors. The 237th and 269th samples are considered ICO samples because of their large ICO‐likeness values. An examination of these samples confirmed that their chemical structures were incorrect. Samples other than the 237th and 269th samples in Figure 7 were considered to contain CO. When these chemical structures were also checked, no errors were found, except for the 237th and 269th samples, despite the large absolute *y* prediction errors. It was confirmed that the proposed ICO‐likeness can properly discriminate between ICO with *x* errors and CO.

**FIGURE 8:**
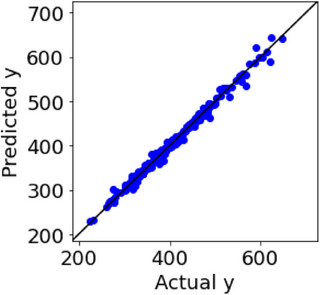
Results of DCV in the compound dataset after correction of chemical structures of the 237th and 269th samples.

After correcting the chemical structures of the 237th and 269th samples, leave‐one‐out cross‐validation was performed using GPR; the results are shown in Figure 8. Compared with Figure 6, the overall number of samples with large absolute *y* prediction errors was reduced. The determinant coefficient *R* ^2^ after DCV improved from 0.971 to 0.992, and the MAE after DCV decreased from 6.5 to 4.3. It was confirmed that the proposed ICO‐likeness method accurately detects the ICO and corrects the *x* errors, thereby improving the prediction accuracy of the model.

## 4 Conclusion

For samples diagnosed as outlier samples, the indicator ICO‐likeness was proposed to determine whether the sample was an ICO or a CO, as shown in Figure 1. ICO‐likeness is calculated based on triple cross‐validation and the mean absolute error; the higher the value of ICO‐likeness, the more likely the target sample is to be an ICO. Data analysis using numerical simulation datasets and a compound dataset with measured boiling points confirmed that the proposed method could distinguish between ICO and CO.

It is worth noting that the proposed ICO‐likeness is not inherently sensitive to the dimensionality of the explanatory variables, as it operates solely on the basis of cross‐validated mean absolute errors. Therefore, whilst the demonstration in this paper uses illustrative datasets, the methodology itself is expected to scale to high‐dimensional settings, assuming that the regression model used (e.g., GPR) maintains its predictive fidelity. Application to larger or noisier datasets remains a valuable future direction.

When an outlier sample is determined to be an ICO, errors in explanatory variables (*x*) and dependent variables (*y*) should be checked first for the sample because the relationship between *x* and *y* is not consistent with the other samples. If neither *x* nor *y* is wrong, it means that the current *x* does not explain *y*; thus, a new *x* should be considered to explain *y* for the ICO. Researchers can examine ICO samples in relation to other samples with similar *x* values but substantially different *y* values. From this comparison, one may explore candidate variables that can discriminate between the ICO sample and these similar‐yet‐different samples, possibly by consulting domain knowledge, performing feature construction or feature engineering.

When an outlier sample is determined to be a CO, because the relationship between *x* and *y* for CO and the other samples is consistent and CO is an outlier compared to the other *y* values, it is expected that exploring the extrapolation from CO in *x* will further improve the *y* values over the existing *y* values using a model constructed with CO. The proposed method is expected to facilitate the design of molecules, materials and processes using machine learning.

## Author Contributions

**Hiromasa Kaneko**: Conceptualization, Data Curation, Formal Analysis, Funding Acquisition, Investigation, Methodology, Project Administration, Resources, Software, Supervision, Validation, Visualization, Writing ‐ Original Draft Preparation, Writing ‐ Review & Editing.

## Conflicts of Interest

The author declares no conflicts of interest.

## Data Availability

Python codes for the proposed method are available at https://github.com/hkaneko1985/dcekit.
